# The prognostic value of myocardial injury in COVID‐19 patients and associated characteristics

**DOI:** 10.1002/iid3.484

**Published:** 2021-07-09

**Authors:** Jian He, Bicheng Zhang, Quan Zhou, Wenjing Yang, Jing Xu, Tingting Liu, Haijun Zhang, Zhiyong Wu, Dong Li, Qing Zhou, Jie Yan, Cuizhen Zhang, Haiyan Qian, Minjie Lu, Xiaoyang Zhou

**Affiliations:** ^1^ State Key Laboratory of Cardiovascular Disease, Department of Magnetic Resonance Imaging, National Center for Cardiovascular Diseases Fuwai Hospital Beijing China; ^2^ Chinese Academy of Medical Sciences and Peking Union Medical College Beijing China; ^3^ Cancer Center Renmin Hospital of Wuhan University Wuhan China; ^4^ Department of Clinical Laboratory Renmin Hospital of Wuhan University Wuhan China; ^5^ Department of Clinical Medicine Wuhan University Wuhan China; ^6^ Department of Cardiology Renmin Hospital of Wuhan University Wuhan China; ^7^ Department of Cardiovascular Surgery Renmin Hospital of Wuhan University Wuhan China; ^8^ Department of Ultrasound Imaging Renmin Hospital of Wuhan University Wuhan China; ^9^ Key Laboratory of Cardiovascular Imaging (Cultivation) Chinese Academy of Medical Sciences Beijing China

**Keywords:** clinical characteristics, coronavirus disease 2019, myocardial injury, prognosis

## Abstract

**Background:**

Since December 2019, coronavirus disease 2019 (COVID‐19) has emerged as an international pandemic. COVID‐19 patients with myocardial injury might need special attention. However, an understanding on this aspect remains unclear. This study aimed to illustrate clinical characteristics and the prognostic value of myocardial injury to COVID‐19 patients.

**Methods:**

This retrospective, single‐center study finally included 304 hospitalized COVID‐19 cases confirmed by real‐time reverse‐transcriptase polymerase chain reaction from January 11 to March 25, 2020. Myocardial injury was determined by serum high‐sensitivity troponin I (Hs‐TnI). The primary endpoint was COVID‐19‐associated mortality.

**Results:**

Of 304 COVID‐19 patients (median age, 65 years; 52.6% males), 88 patients (27.3%) died (61 patients with myocardial injury, 27 patients without myocardial injury on admission). COVID‐19 patients with myocardial injury had more comorbidities (hypertension, chronic obstructive pulmonary disease, cardiovascular disease, and cerebrovascular disease); lower lymphocyte counts, higher C‐reactive protein (CRP; median, 84.9 vs. 28.5 mg/L; *p* < .001), procalcitonin levels (median, 0.29 vs. 0.06 ng/ml; *p* < .001), inflammatory and immune response markers; more frequent need for noninvasive ventilation, invasive mechanical ventilation; and was associated with higher mortality incidence (hazard ratio [HR] = 7.02; 95% confidence interval [CI], 4.45–11.08; *p* < .001) than those without myocardial injury. Myocardial injury (HR = 4.55; 95% CI, 2.49–8.31; *p* < .001), senior age, CRP levels, and novel coronavirus pneumonia types on admission were independent predictors to mortality in COVID‐19 patients.

**Conclusions:**

COVID‐19 patients with myocardial injury on admission is associated with more severe clinical presentation and biomarkers. Myocardial injury and higher Hs‐TnI are both strongest independent predictors to COVID‐19‐related mortality after adjusting confounding factors.

## Graphical abstract

COVID patients with myocardial injury on admission is associated with more severe clinical presentation and biomarkers. Myocardial injury (hazard ratio = 4.55; *p* < 0.001) and higher HsTNI are both strongest independent predictors to COVID‐related mortality after adjusting confounding factors.

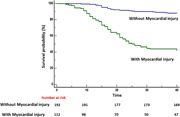

## 1 BACKGROUND

Coronavirus disease 2019 (COVID‐19) is a newly recognized infection which was first reported in Wuhan, China.^1^ Since the beginning of the outbreak, COVID‐19 has emerged as a pandemic globally,^2^ and the number of cases is rising at an exponential rate.^3^ As of June 18, 2021, there have been a total of more than 176,693,988 laboratory‐confirmed cases of COVID‐19 globally, and it poses a great threat to public health in the world as evidenced by 3,830,304 deaths.^4^ COVID‐19 is caused by the severe acute respiratory syndrome coronavirus‐2 (SARS‐CoV‐2). The clinical spectrum appears to be very wide, including asymptomatic infection, mild upper respiratory disease, and severe viral pneumonia with respiratory failure, and even death.^5^ The condition of some patients with COVID‐19 may deteriorate rapidly, particularly in older patients with underlying comorbidities including cardiovascular disease.^6^ Furthermore, SARS‐CoV‐2 can affect the cardiovascular system in multiple ways, increasing morbidity in patients with potential cardiovascular disease and causing myocardial injury and dysfunction.^3^ High‐sensitivity troponin I (Hs‐TnI) provides the potential to earlier identify myocardial injury and assists treatment.^7^ Some studies provide insights into the incidence of cardiac complications associated with SARS‐CoV‐2,^8^, ^9^ while imaging manifestations, cytokine levels, and the prognostic value of cardiovascular risk factors in COVID‐19 patients are poorly understood. We aimed to comprehensively define clinical characteristics, laboratory results, outcomes, and management strategies of COVID‐19 patients, then to find whether there is an association of myocardial injury and other biomarkers with mortality. This study may also provide clues to potential mechanisms associated with myocardial injury.

## 2 METHODS

### 2.1 Study design and patients

In this study, we retrospectively enrolled 320 COVID‐19 patients admitted to the Renmin Hospital of Wuhan University from January 11, 2020 to March 25, 2020 with approval from the Research Ethics Committee of the Renmin Hospital of Wuhan University, Wuhan, China (approval number: WDRY2020‐K038). Sixteen cases without significant biomarkers, including Hs‐TnI and creatinine kinase–myocardial band (CK‐MB) levels, were excluded. Thus, a total of 304 patients were finally included in the study. The confirmed diagnosis of COVID‐19 was defined as a positive result by using real‐time reverse‐transcriptase polymerase‐chain‐reaction (RT‐PCR) detection for routine nasal and pharyngeal swab specimens or anti‐SARS‐CoV‐2 antibody assay.

### 2.2 Data colle**c**tion

The demographic characteristics, clinical data, and results of cardiac biomarkers were obtained from the hospital's electronic medical records according to previously designed standardized data collection forms. The date of symptom onset, initial diagnosis of COVID‐19, and death were recorded. The clinical features of symptoms and signs and comorbidities were collected on admission. Laboratory analyses included complete blood count, hepatic function, kidney function, coagulation function, C‐reactive protein (CRP), lactate dehydrogenase (LDH), myocardial enzymes, procalcitonin (PCT), electrolytes test, and status of other viral infection. Cardiac biomarkers including N‐terminal pro‐B‐type natriuretic peptide (NT‐proBNP), Hs‐TnI, CK‐MB, and myoglobin were measured on admission and during hospitalization. Routine bacterial and fungal examinations were also performed. Radiologic assessments included digital radiography (DR) and/or computed tomography (CT). Two researchers collected and checked the final database.

### 2.3 Defini**t**ions

Fever was defined as an axillary temperature of 37.5°C or higher.^10^ The date of onset was defined as the day when any symptom was noticed. Myocardial injury was defined as serum levels of Hs‐TnI were above the 99th percentile upper reference limit at admission (0.04 ng/ml, measured in the laboratory of Renmin Hospital of Wuhan University).^11^ The novel coronavirus pneumonia (NCP) types (mild, common, severe, critically severe) was defined according to the diagnostic and treatment programme for SARS‐CoV‐2 issued by Chinese National Health Committee (version 7).^12^ The primary endpoint was COVID‐19‐associated death. Hospital discharge was allowed after the relieved clinical symptoms, normal body temperature for at least 3 days, significant improvement in radiological findings, and at least two consecutive negative results shown by RT‐PCR for COVID‐19.^12^

### 2.4 Statistical a**n**alysis

Categorical variables are shown as frequency rates or percentages. Continuous variables as mean ±* SD* or median (interquartile range [IQR]) as appropriate. Student's *t* test, Mann–Whitney test, *χ* ^2^ test or Fisher's exact test was used where appropriate to assess the difference between different cohorts. The Pearson correlation coefficient and Spearman rank correlation coefficient were used for correlation analysis. Survival curves were plotted using the Kaplan–Meier method between patients with and without myocardial injury. Univariate and multivariate Cox regression models were used to determine the independent risk factors for COVID‐19‐associated death in the hospital. The NCP types was listed as rank variable. Statistical analysis was performed with SPSS, version 21.0 (IBM). A two‐sided *p* < .05 was considered statistically significant.

## 3 RESULTS

A total of 304 hospitalized patients with COVID‐19 were consecutively enrolled in our study, including 96 patients (31.6%) with myocardial injury and 208 patients (68.4%) without myocardial injury. The demographic and clinical characteristics of the patients are listed in Table 1. On admission, over half of the COVID‐19 patients (198, 65.1%) had comorbidities, including hypertension (42.8%), diabetes (16.4%), and cardiovascular disease (16.1%), like coronary artery disease (10.5%), arrythmia (4.3%), and cardiomyopathy (0.7%). COVID‐19 patients with myocardial injury were older, more males, and more likely to have pre‐existing comorbidities, and were associated with more severe presentation (critically severe, 26.0% vs. 7.2%; all *p* < .001).

**Table 1:** Clinical characteristics and complications of COVID‐19 patients

Characteristic	All patients (*n* = 304)	Myocardial injury		*p* value
		With (*n* = 96)	Without (*n* = 208)	
Male (%)	160 (52.6)	61 (63.5)	99 (47.6)	**.010**
Age, median (IQR)	65.0 (54.0–74.0)	70.5 (60.1–79.0)	62.0 (52.0–69.0)	**<.001**
NCP types (%)				
Mild/common	117 (38.5)	22 (22.9)	95 (45.7)	**<.001**
Severe	147 (48.4)	49 (51.0)	98 (47.1)	
Critically severe	40 (13.2)	25 (26.0)	15 (7.2)	
Comorbidities (%)	198 (65.1)	76 (79.2)	122 (58.7)	**<.001**
Diabetes	50 (16.4)	17(17.7)	33 (15.9)	.687
Hypertension	130 (42.8)	52 (54.2)	78 (37.5)	**.006**
COPD	21 (6.9)	12 (12.5)	9 (4.3)	**.009**
Cardiovascular disease	49 (16.1)	27 (28.1)	22 (10.6)	**<.001**
Coronary artery disease	32 (10.5)	18 (18.8)	14 (6.7)	**.002**
Cerebrovascular disease	21 (6.9)	13 (13.5)	8 (3.8)	**.002**
Kidney disease	12 (3.9)	4 (4.2)	8 (3.8)	1
Hepatic disease	8 (2.6)	3 (3.1)	5 (2.4)	.711
Cancer, auto‐immune disease	32 (10.5)	11 (11.5)	21 (10.1)	.719

*Note*: *p* values are calculated by Student's *t* test, Mann–Whitney *U* test, or *χ* ^2^ test as appropriate. *p* values less than .05 was statistical significant (Boldface).

Abbreviations: COPD, chronic obstructive pulmonary disease; IQR, interquartile range; NCP, novel coronary pneumonia.

**Figure 1:**
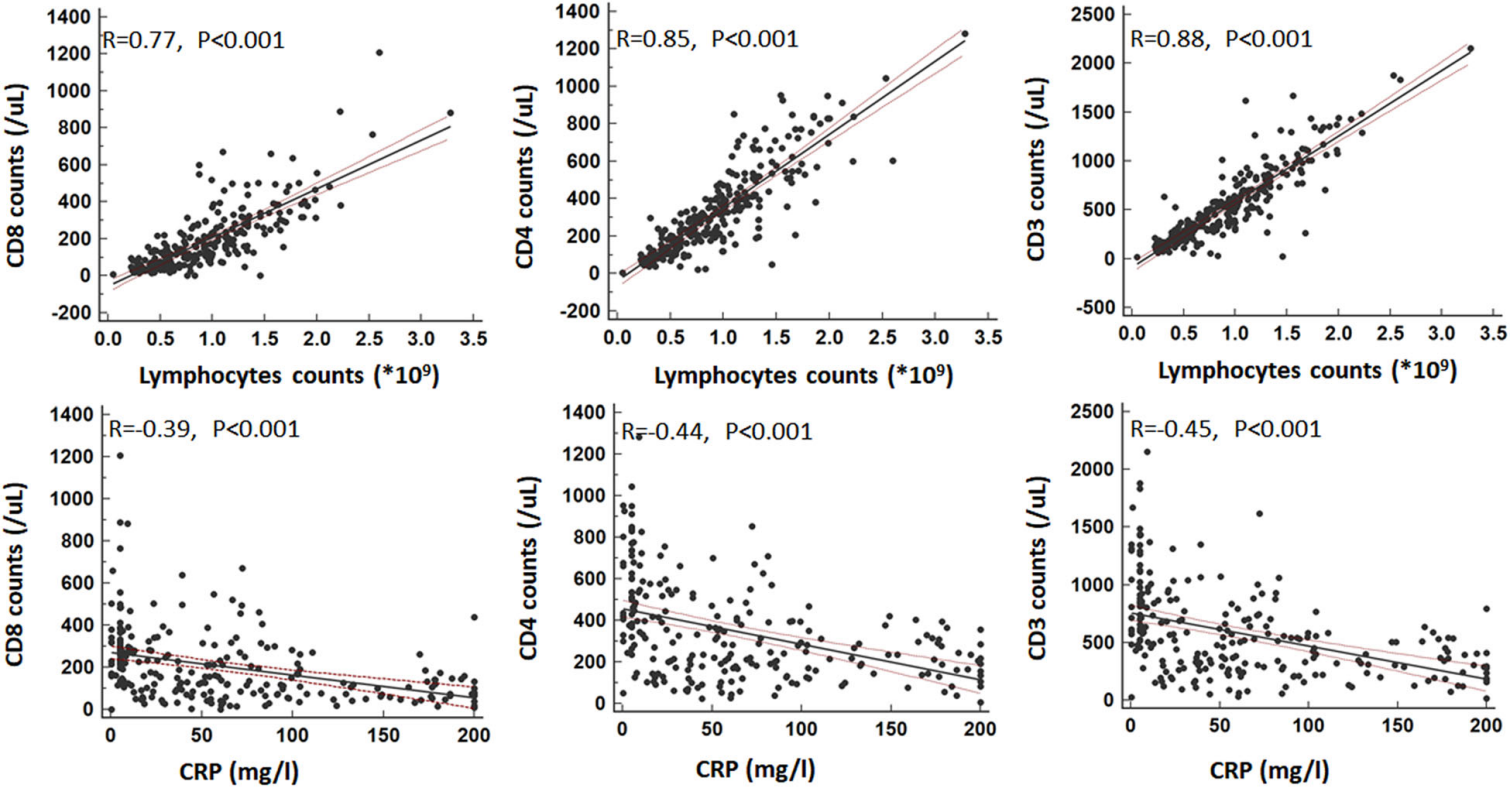
Associations between CD3, CD4, CD8, and lymphocytes biomarkers. Strong correlations were found between CD3, CD4, CD8, and lymphocytes biomarkers. CD, cluster of differentiation of T cells

**Figure 2:**
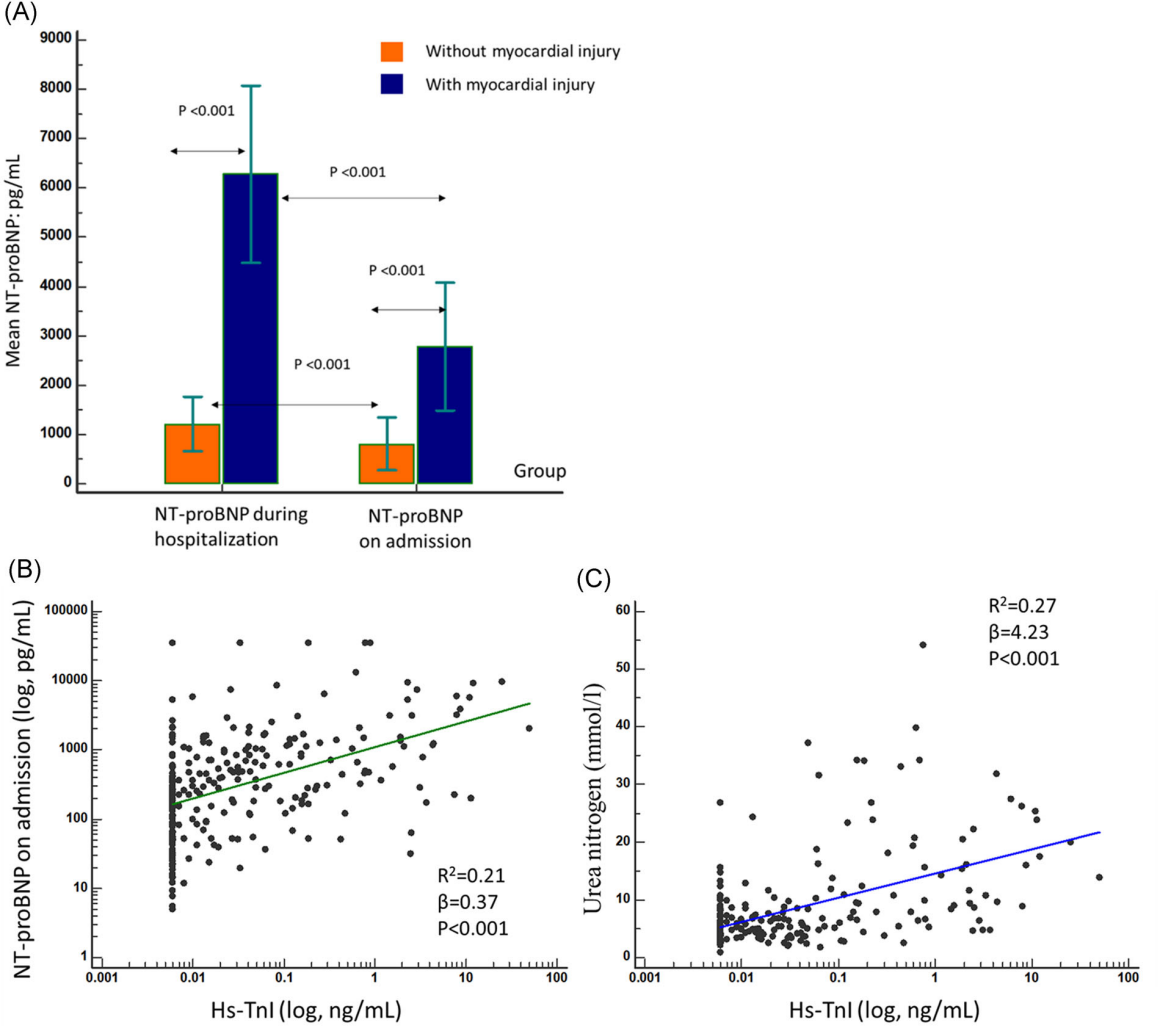
Progression of NT‐proBNP in COVID‐19 patients on admission and during hospitalization, and correlation between serum high‐sensitivity TnI and NT‐proBNP, urea nitrogen. When Hs‐TnI levels was <0.006 ng/ml, it was recorded as 0.006 ng/ml at convenience. Hs‐TnI, high‐sensitivity troponin I; NT‐proBNP, N‐terminal pro‐B‐type natriuretic peptide

On admission, the median leukocyte counts, neutrophil counts, platelet counts, hemoglobin, immunoglobulin G (IgG), IgM, IgA, and complement 3 (C3) levels were all within the normal range in both groups (Table 2). However, significant differences were noted in neutrophil and platelet counts between the two groups (*p* < .001). Comparing with patients without myocardial injury, patients with myocardial injury had even lower CD3, CD4, CD8 counts, higher IL‐6, CRP, and PCT levels (all *p* < .001), and CD3, CD4, CD8 counts showed strong correlations (*R* > 0.77) with lymphocytes counts and moderate correlations (*R*: −0.39 to −0.45) with CRP levels (Figure 1). Log‐transformed serum Hs‐TnI levels in patients with COVID‐19 correlated significantly with both log‐transformed serum NT‐proBNP levels (*β* = 0.37; *p* < .001) and serum urea nitrogen levels (*β* = 4.23; *p* < .001) (Figure 2).

**Table 2:** Laboratory examinations and radiographic presentation in COVID‐19 patients

Characteristic	Normal range	All patients (*n* = 304)	Myocardial injury		*p* value
			With (*n* = 96)	Without (*n* = 208)	
Immunologic markers, median (IQR)					
Leucocyte counts (10^9^/L)	3.5–9.5	6.3 (4.3–8.1)	7.1 (5.0–10.1)	6.0 (4.4–7.8)	.058
Neutrophil counts (10^9^/L)	1.8–6.3	4.9 (3.0–7.4)	6.7 (4.2–10.3)	4.4 (2.8–6.4)	**<.001**
Lymphocyte counts (10^9^/L)	1.1–3.2	0.9 (0.6–1.3)	0.6 (0.4–1.0)	1.0 (0.7–1.3)	**<.001**
Platelet counts (10^9^/L)	130–175	200.0 (145.0–258.0)	151.5(105.0–237.2)	210.0 (165.5–264.0)	**<.001**
Hemoglobin (g/L)	125–350	123.0 (111.3–136.0)	122.0 (110.0–137.0)	123.0 (113.0–135.2)	.872
CD3 counts (per µl)	723–2737	502.0 (275.8–765.0)	317.5 (177.3–591.0)	539.5 (324.0–862.5)	**<.001**
CD4 counts (per µl)	404–1612	287 (161.5–464.5)	193.5 (98.8–322.8)	340.5 (201.8–528.3)	**<.001**
CD8 counts (per µl)	220–1129	152.0 (73.0–278.0)	77 (40.8–161.5)	191.0 (106.0–304.0)	**<.001**
CD4/CD8 ratio	0.9–2.0	1.8 (1.3–1.7)	2.0 (1.3–3.4)	1.8 (1.3–2.7)	.127
IgG (g/L)	7.0–16.0	12.3 (10.2–15.4)	13.7 (11.2–16.6)	11.7 (9.6–14.6)	**.003**
IgM (g/L)	0.4–2.3	1.0 (0.7–1.3)	1.0 (0.7–1.3)	1.0 (0.7–1.2)	.143
IgA (g/L)	0.7–4.0	2.4(1.8–3.3)	2.8 (2.1–3.8)	2.2 (1.7–3.0)	**.002**
C3 (g/L)	0.9–1.8	1.0 (0.9–1.1)	1.0 (0.8–1.1)	1.0 (0.9–1.2)	**<.001**
CK‐MB (ng/ml)	0–5	1.2 (0.7–2.6)	4.2 (1.9–8.3)	0.9 (0.7–1.5)	**<.001**
Cardiac, hepatic, and kidney injury markers, median (IQR)					
Myoglobin (µg/L)	0–110	49.1 (27.4–130.9)	177.4 (82.5–765.7)	35.0 (25.0–59.7)	**<.001**
Hs‐TnI (ng/ml)	0–0.04	<0.006 (<0.006–0.068)	0.22 (0.09–1.83）	<0.006 (<0.006–0.011)	**<.001**
NT‐proBNP^a^ (pg/ml)	0–300	285.8 (86.7–835.8)	799.7 (267.7–1719.0)	220.1 (54.0–456.5)	**<.001**
NT‐proBNP^b^ (pg/ml)	0–300	647.8 (237.2–1996.3)	2543.0 (953.0–9022.0)	389.0 (141.0–1046.0)	**<.001**
LDH (U/L)	100–300	266 (202.8–413.3)	433.5 (306.5–677.5)	221.0 (188.0–284.0)	**<.001**
ALT (U/L)	9–50	24.0 (17.0–46.0)	27.0 (18.0–48.0)	24.0 (16.8–43.3)	.662
AST (U/L)	15–40	27.0 (19.0–43.5)	42.0 (24.0–65.0)	23.5 (17.3–32.0)	**<.001**
ALP (U/L)	90–130	71.0 (56.4–94.0)	75.0 (58.8–105.0)	69.0 (56.0–90.3)	.219
ALB (g/L)	40–55	37.0 (33.5–40.0)	33.8 (30.1–37.1)	38.7 (35.8–41.0)	**<.001**
Urea (mmol/L)	3.6–9.5	5.4 (3.8–9.0)	9.5 (5.4–19.4)	4.6 (3.5–6.5)	**<.001**
Creatinine (mmol/L)	57–111	58.0 (48.0–79.0)	71.0 (53.0–126)	56.0 (46.0–70.8)	**<.001**
Potassium (mmol/L)	3.5–5.5	4.2 (3.8–4.5)	4.1 (3.6–4.6)	4.3 (3.9–4.5)	.255
Sodium (mmol/L)	135–155	142.0 (139.0–146.0)	141.0 (138.0–146.0)	142.0 (139.0–146.0)	.542
Inflammation markers, median (IQR)					
PCT (ng/ml)	<0.1	0.10 (0.05–0.32)	0.29 (0.10–1.09)	0.06 (0.04–0.14)	**<.001**
C‐reactive protein (mg/L)	0–10	51.3 (10.9–104.0)	84.9 (53.7–173.8)	28.5 (5.7–82.2)	**<.001**
IL‐6 (pg/ml)	<10	10.5 (6.1–26.5)	23.5 (10.7–98.1)	9.0 (5.8–20.6)	**<.001**
Coagulation markers, median (IQR)					
PT (s)	9–13	12.4 (11.5–13.5)	13.4 (12.2–14.4)	12.1 (11.3–13.1)	**<.001**
APTT (s)	25–31.3	28.6 (26.2–31.5)	29.2 (27.7–33.2)	28.2 (25.9–31.0)	**<.001**
D‐dimer (mg/L)	0–0.55	2.5 (0.7–13.8)	7.0 (1.9–21.7)	1.6 (0.6–8.2)	**<.001**

Abbreviations: ALB, albumin; ALP, alkaline phosphatase; ALT, alanine aminotransferase; APTT, active partial thromboplastin time; AST, aspartate aminotransferase; C3, complement 3; CD, cluster of differentiation; CK‐MB, creatinine kinase–myocardial band; ECG, electrocardiogram; Hs‐TnI, high‐sensitivity troponin I; Ig, immunoglobulin; IL‐6, interleukin 6; IQR, interquartile range; LDH, lactate dehydrogenase; NT‐proBNP, N‐terminal pro‐B‐type natriuretic peptide; PCT, procalcitonin; PT, prothrombin time.

*Note*: *p* values are calculated by Student's *t* test, Mann–Whitney *U* test, or *χ* ^2^ test as appropriate. *p* values less than 0.05 was statistical significant (Boldface).

a NT‐proBNP levels on admission.

b NT‐proBNP levels during hospitalization.

Regarding the cardiac, hepatic, renal, and coagulation function (Table 2), patients with COVID‐19 had increased Hs‐TnI, PCT, and D‐dimer levels on admission and NT‐proBNP during hospitalization compared with the normal reference values. Patients with myocardial injury showed elevated myoglobin, LDH, PCT, and D‐dimer levels (all *p* < .001) compared with patients without myocardial injury. Figure 2 shows the dynamic change of NT‐proBNP of COVID‐19 patients from admission to hospitalization.

The imaging manifestations of the 304 patients with COVID‐19 were listed in Table S1. One hundred and thirty‐five patients (44.4%) underwent examination of electrocardiogram (ECG) after admission, and 83 of 135 ECGs (61.5%) indicated cardiac abnormalities, including T‐wave depression and inversion, ST‐segment depression, and atrioventricular block. Thirty‐four patients (11.2%) underwent examination with echocardiography and 27 patients (79.4%) showed abnormalities, and the more common abnormalities are cardiac diastolic dysfunction, and other complex abnormalities (tricuspid regurgitation). All patients underwent CT or DR examinations and 221 (72.6%) patients presented with pulmonary abnormalities including ground‐glass opacities or consolidation (Figure S1). There was no statistically significant difference in the other imaging findings between patients with or without myocardial injury, only the patients with complex echo abnormalities had significant difference.

Oxygen treatment was provided to 206/304 patients (67.8%). Sixty‐two (20.4%) patients received noninvasive ventilation, and 10.2% of the patients (31 patients) were placed in mechanical ventilation. The proportion treated with antiviral therapy was the highest (304, 100%), followed by high‐dose glucocorticoid therapy (142, 46.7%), intravenous immunoglobulin therapy (122, 40.1%), antibiotic therapy (118, 38.8%) and hemoperfusion (20, 6.6%). Only 8 patients (2.6%) among all participants were given plasmapheresis therapy. Overall, 100 patients (27.3%) had kidney injury during hospitalization, and 65 patients (21.4%) had hepatic injury.

**Figure 3:**
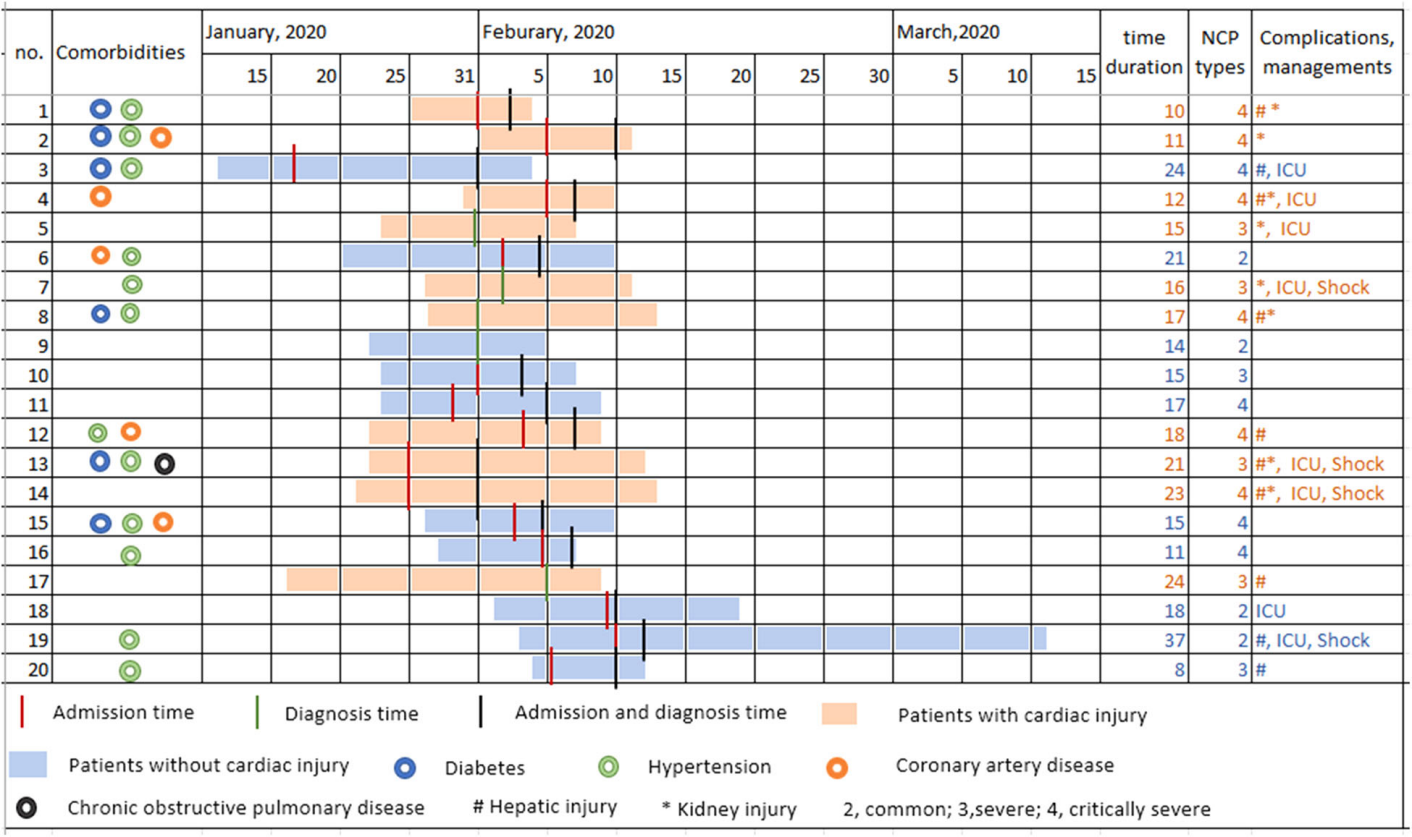
Days from symptom onset to death of COVID‐19 patients with or without myocardial injury and their comorbidities, complications, managements. Comparison of days from symptom onset to death of typical 20 patients with COVID‐19 with myocardial injury (10 patients) or without myocardial injury (10 patients) randomly selected from dead patients. Patients with myocardial injury were more likely to have comorbidities, severe presentation, and complications, shock and ICU managements. However, no significant difference was found for time duration from symptom onset to death in two groups. This figure tells us that myocardial injury may be associated with more severe presentation. ICU, intensive care unit; NCP, novel coronary pneumonia

Compared with those without myocardial injury, more COVID‐19 patients with myocardial injury required oxygen inhalation, noninvasive ventilation, invasive mechanical ventilation, antibiotic treatment, and hemoperfusion therapy (all *p* < .001) (Table 3). However, intravenous immunoglobulin treatment (24.0% vs. 47.6%; *p* < .001) was lower in patients with myocardial injury. The comparison of typical managements, comorbidities, complications, and time duration from symptom onset to death were made in 20 random patients with myocardial injury or not (Figure 3), indicating that myocardial injury may be associated with more severe presentation.

**Table 3:** Managements and clinical outcomes of COVID‐19 patients

Characteristic	All patients (*n* = 304)	Myocardial injury		*p* value
		With (*n* = 96)	Without (*n* = 208)	
Managements, *n* (%)				
Oxygen inhalation	206 (67.8)	85 (88.5)	121 (58.2)	**<.001**
Noninvasive ventilation	62 (20.4)	35 (36.5)	27 (13.0)	**<.001**
Invasive mechanical ventilation	31 (10.2)	20 (20.8)	11 (5.3)	**<.001**
Immunoglobulin	122 (40.1)	23 (24.0)	99 (47.6)	**<.001**
Antiviral	304 (100)	96 (100)	208 (100)	‐
Antibiotic	118 (38.8)	65 (67.7)	53 (25.5)	**<.001**
Glucocorticoids	142 (46.7)	44 (45.8)	98 (47.1)	.835
Hemoperfusion	20 (6.6)	12 (12.5)	8 (3.8)	**.005**
Plasmapheresis	8 (2.6)	5 (5.2)	3 (1.4)	.114
Clinical outcomes, *n* (%)				
Death	88 (27.3)	61 (63.5)	27 (13.0)	**.005**
In hospital	83 (28.9)	11 (11.5)	72 (34.6)	
Discharge	133 (43.8)	24 (25.0)	109 (52.4)	

*Note*: *p* values are calculated by Student's *t* test, Mann–Whitney *U* test, or *χ* ^2^ test as appropriate. *p* values less than 0.05 was statistical significant (Boldface).

**Figure 4:**
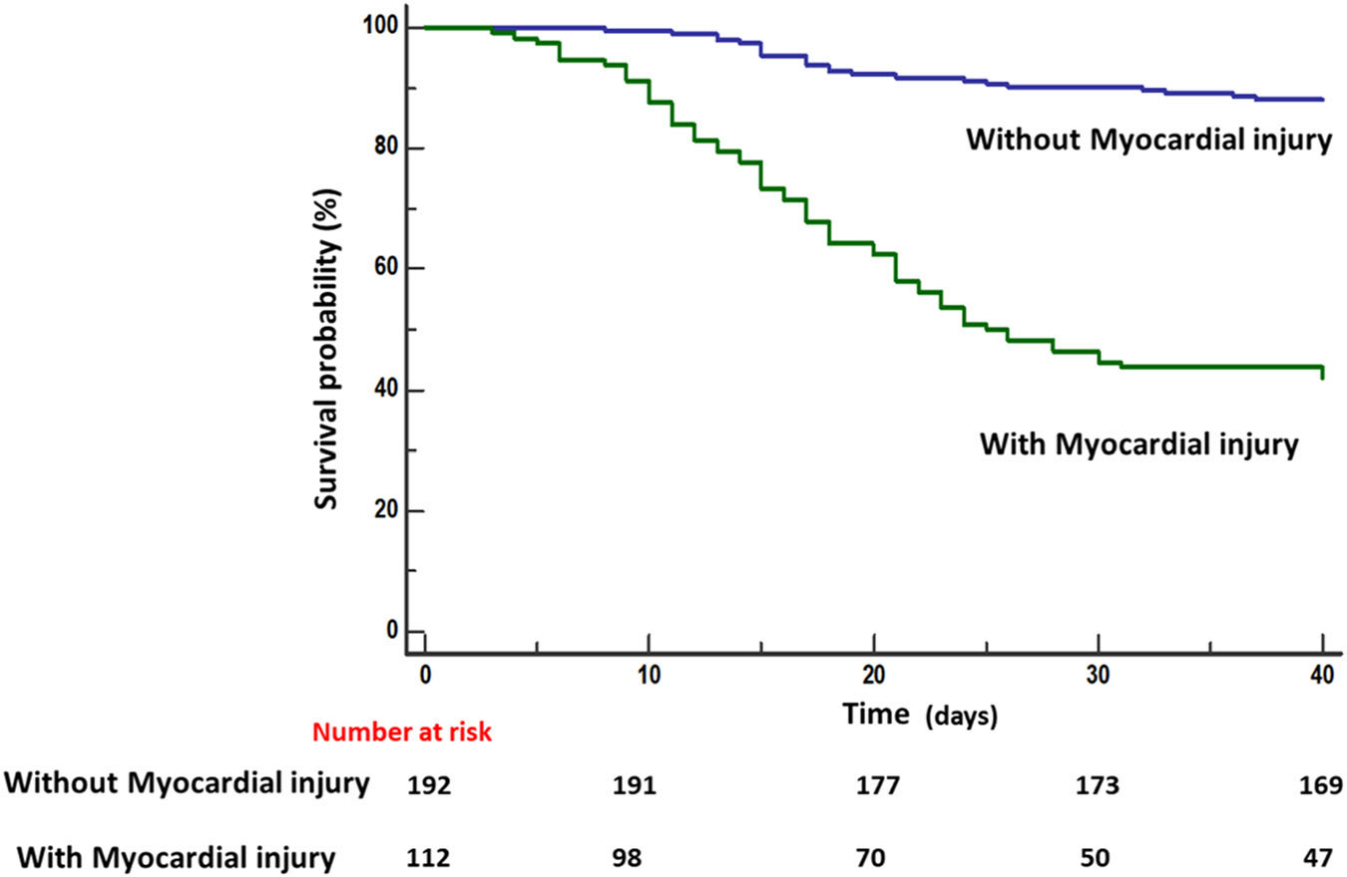
Mortality during hospitalization between patients with versus without myocardial injury. Kaplan–Meier survival curves for mortality from symptom onset to follow‐up date

During the median durations for about 45.4 days from onset of symptoms to follow‐up (range: 3–84 days), a total of 88/304 patients (27.3%) died, among which 61 patients had myocardial injury, while 27 patients did not have myocardial injury (Table 3). In the univariable analysis, the mortality rate was significantly higher in patients with myocardial injury (63.5% vs. 13.0%; *p* < .001; Table 3). The Kaplan–Meier survival curves indicated significant survival differences between the patients with or without myocardial injury (*p* < .001; Figure 4). The multivariate Cox proportional hazard regression model showed significantly higher risk of death in patients with myocardial injury than in those without myocardial injury from symptom onset (hazard ratio [HR], 4.55; 95% confidence interval [CI], 2.49–8.31; *p* < .001) to primary endpoint, after adjusting for age, sex, pre‐existing comorbidities, CRP levels, D‐dimer levels, NCP types (Table 4). Under this Cox regression model, senior age (HR, 2.01; 95% CI, 1.03–3.92; *p* = .04), CRP levels (HR, 1.01; 95% CI, 1.00–1.01; *p* = .001), and NCP types (*p* = .007) were other independent risk factors for mortality with COVID‐19. In addition, Cox regression model with Hs‐TnI as continuous variable showed that higher Hs‐TnI was also associated with mortality (HR, 3.33; 95% CI, 1.96–5.66; *p* < .001) (Table S2).

**Table 4:** Multivariate Cox regression analysis on the risk factors associated with mortality in patients with COVID‐19 from symptom onset

Factors	Univariate analysis		Cox regression model	
	Hazard ratio (95% CI)	*p* value	Hazard ratio (95% CI)	*p* value
Age, >65 years	3.79 (2.32–6.20)	**<.001**	2.01 (1.03–3.92)	**.04**
Sex	0.53 (0.34–0.82)	**.005**		
Hypertension	2.05 (1.34–3.13)	**.001**		
COPD	2.95 (1.64–5.32)	**<.001**		
Chronic heart disease	2.16 (1.34–3.47)	**.002**		
Cerebrovascular disease	3.10 (1.72–5.59)	**<.001**		
Myocardial injury	7.02 (4.45–11.08)	**<.001**	4.55 (2.49–8.31)	**<.001**
CRP	1.01 (1.01–1.02)	**<.001**	1.01 (1.00–1.01)	**.001**
NT‐proBNP on admission	1.00 (1.00–1.00)	.082		
PCT	1.00 (1.00–1.02)	.768		
D‐dimer	1.01 (1.00–1.01)	**.003**		
NCP types		**<.001**		**.007**
Severe‐common	3.89 (1.95–7.76)	**<.001**	2.18 (0.92–5.15)	.075
Critically severe‐common	18.42 (9.06–37.41)	**<.001**	4.33 (1.65–11.36)	**<.001**

*Note*: *p*‐values by Cox regression analyses. PCT, CRP, and NT‐proBNP on admission, D‐dimer were performed as continuous variables. *p* values less than 0.05 was statistical significant (Boldface).

Abbreviations: CI, confidence interval; COPD, chronic obstructive pulmonary disease; NCP, novel coronary pneumonia; NT‐proBNP, N‐terminal pro‐B‐type natriuretic peptide; PCT, procalcitonin.

## 4 DISCUSSION

This study summarizes the clinical characteristics, laboratory, cardiac, and radiographic findings in a large cohort of 304 hospitalized COVID‐19 patients, and provides novel information of the prognostic value of pre‐existing comorbidities and myocardial injury. Myocardial injury, senior age, NCP types, and CRP levels were independently associated with higher risk of mortality during hospitalization. The myocardial injury was probably associated with inflammation response. The prognostic value of elevated Hs‐TnI in patients with COVID‐19 should be of great interest to a broad readership, as a simple blood biomarker test was such a strong predictor of mortality and that sophisticated, expensive, and time‐consuming cardiac testing with CT and magnetic resonance imaging are not needed.

With the high infectivity, COVID‐19 has managed to supersede severe acute respiratory syndrome (SARS) in 2003 and Middle East respiratory syndrome (MERS) in 2012 in terms of death toll.^13^ The median age was 65 years in our study, greater than previous studies (55.6 and 49 years),^9^, ^14^ mainly due to the severe clinical types and more comorbidities in our patient cohort tending to be older. This is supported by the evidence that the median age of patients is higher in group with myocardial injury than those without (70.5 vs. 62.0 years). COVID‐19 patients with myocardial injury were prone to develop severe NCP types (77.0%), and more likely to have pre‐existing comorbidities (79.2% vs. 58.7%), like hypertension, cardiovascular disease and cerebrovascular disease, also confirmed by other studies.^9^, ^15^ Li et al.^16^ analyzed six studies involving 1527 COVID‐19 patients with previous cardiovascular metabolic diseases, and indicated that this patient group may face a greater risk of developing into the severe condition and that comorbidities can also greatly affect the prognosis of the COVID‐19 patients.

In our study, COVID‐19 patients with myocardial injury had obviously elevated myoglobin, and NT‐proBNP levels, providing independent corroborating evidence of myocardial injury.^17^ In addition, the correlations between Hs‐TnI levels and NT‐proBNP levels with urea nitrogen levels indicated multiorgan injury along with myocardial injury and worth early monitoring. In prior studies, no significant deviations in coagulation function from the normal range were found but some patients still presented with coagulation dysfunction.^18^, ^19^ Our study further validated this result, especially in patients with myocardial injury or in critically severe and severe type. Hence, we speculate that coagulation may be not a very important pathophysiological process in all patients with COVID‐19. Only some critically ill patients demonstrated abnormal coagulation function and multiple organ dysfunction at the end stage. COVID‐19 patients with myocardial injury need active treatments to delay or reverse the progression of disease, supported by the fact that the clinical presentation and the severity of COVID‐19, as well as more comorbidities in patients with myocardial injury group. However, patients without myocardial injury received more intravenous immunoglobulin therapy than patients with myocardial injury, and this may be attributable to rapid progression of COVID‐19 disease in the later cohort, which makes it too late to use intravenous immunoglobulin therapy.

Two prior studies indicated that there was a strong correlation between myocardial injury and prognosis of COVID‐19 patients during hospitalization.^8^, ^9^ Adding to previous reports, our study further confirmed that myocardial injury, senior age, CRP levels, and NCP types are all independent prognostic indicators of mortality in COVID‐19 patient. Among these risk factors, myocardial injury, determined by serum Hs‐TnI, was the strongest both as dichotomous and continuous variable. The Hs‐TnI marker can be an ally for earlier identifying myocardial injury, rather than myocardial infarction, thus guiding timely intervention.^7^

To date, the exact mechanism of cardiac involvement in COVID‐19 remains under investigation. Current studies suggested immune change in patients with MERS,^20^ SARS^21^ and influenza,^22^ especially changes in peripheral blood T cells, which may contribute to understanding the characteristics, diagnosis, monitoring, prevention, and treatment of the disease. Many investigations have already reported that the pathophysiology of myocardial injury in COVID‐19 patients may be linked to dysregulation of immune response, presenting with lower lymphocyte counts, higher leukocytes counts, and significantly reduced CD4+ and CD8+ T lymphocytes levels.^23^, ^24^, ^25^ In our study, the CD3, CD4, CD8 counts correlated well with lymphocytes counts and CRP levels, and elevated inflammation reaction (CRP) and suppressive immune response (CD4+, CD8+) were observed in COVID‐19 patients at admission. In addition, traditional cardiovascular risk factors such as diabetes and hyperlipidemia impact immune function, and conversely, dysregulated immunologic status corresponds with elevated risk of incident cardiovascular disease.^26^ Another possible mechanism is the direct invasion via angiotensin converting enzyme 2 (ACE2) receptors in cardiovascular system.^3^, ^27^ In our study, we not only found that COVID‐19 patients with comorbidities were associated with higher mortality, but also the CRP levels are significantly associated with mortality in multivariate Cox analysis, which indicated there would be inflammation response and this was associated with worse outcomes. In addition, IL‐6 cytokines were elevated in COVID‐19 patients with myocardial injury, justifying the emergence of severity of inflammation, also immune‐related markers, like CD3, CD4, CD8 molecules counts and lymphocyte counts were all reduced, more significant in patients with myocardial injury. This was consistent with a previous study, which indicated that lymphopenia was an independent risk factor of myocardial injury in the setting of COVID‐19.^28^ It was reasonable to presume that invasive coronavirus may dysregulate the immune system, and further lead to severe damage to myocardial tissues. The data from all these studies taken together, suggest that clinicians should consider myocardial injury on presentation, NCP types and inflammation and immune dysregulation when caring for COVID‐19 patients. The exact mechanism of myocardial injury merits further investigation, but the findings presented here highlight the prognostic value in identifying myocardial injury with noninvasive biomarker testing on admission in COVID‐19 patients and raise the possibility that providers should consider close management of immune response, inflammation, and comorbidities in COVID‐19 hospitalized patients.

We acknowledge some limitations in our study. First, this was a retrospective, single‐center study of patients admitted to hospital; multi‐center investigations for a larger cohort would be better to assess the clinical characteristics and confirm the outcomes of myocardial injury after infection with COVID‐19. Second, because of the logistical limitations at the onset of these emerging infections in Wuhan, some data, such as inflammation biomarker and imaging data were lacking on admission, which limits further confirmation of potential mechanisms of myocardial injury. Third, the data in this study permit a preliminary assessment of the clinical course and outcomes of patients with COVID‐19. The causes of death may involve multiple organ dysfunction in most cases, and it is difficult to differentiate the myocardial injury as the main and direct cause in an individual case. Long‐term observation and prospective study design on the effectiveness of treatments specific for the myocardial injury are needed.

## 5 CONCLUSIONS

In conclusion, myocardial injury is common in patients hospitalized with COVID‐19. Patients with myocardial injury had more severe presentation and complex comorbidities. Furthermore, myocardial injury is independently associated with increased in‐hospital mortality in patients with COVID‐19.

## ETHICS STATEMENT

This study obtained approval from the Research Ethics Committee of the Renmin Hospital of Wuhan University, Wuhan, China (approval number: WDRY2020‐K038). Written consent was obtained from all participants.

## AUTHOR CONTRIBUTIONS

**Jian He, Bicheng Zhang, Quan Zhou, Wenjing Yang, Jing Xu, Tingting Liu, Haijun Zhang, Zhiyong Wu, Dong Li, Qing Zhou, Jie Yan, Cuizhen Zhang**: Data curation; formal analysis. **Bicheng Zhang, Quan Zhou, JZ**: Data curation; resources; investigation; **Jian He, Wenjing Yang, Jing Xu**: Writing; methodology. **Haiyan Qian, Minjie Lu, Xiaoyang Zhou**: Methodology, project administration, conceptualization. **Minjie Lu and Xiaoyang Zhou**: Conceptualization; validation; supervision; funding acquisition. All authors have read and approved the manuscript.

## Supporting information

Supplementary information.

## Data Availability

The data sets used and/or analysed during the current study are available from the corresponding author on reasonable request.
