# Supplementary material for: The prognostic value of myocardial injury in COVID‐19 patients and associated characteristics
Source: Immun Inflamm Dis. 2021 Jul 9;9(4):1358–69. doi: 10.1002/iid3.484 (PMC8427070; doi:10.1002/iid3.484)
Supplement: Supplementary file 1 — Supplementary information. [file IID3-9-1358-s001.docx]

**Supplementary Files**

Table S1: Imaging abnormalities in patients with COVID-19.

| Parameters, n (%) | All patients | Myocardial Injury | | P value |
| --- | --- | --- | --- | --- |
|  |  | With | Without |  |
| **ECG abnormalities** | 83(61.5), n=135 | 25 (59.5), n=42 | 58 (64.5), n=93 | 0.753 |
| Sinus arrhythmia | 11 (8.1) | 2 (4.8) | 9 (9.7) | 0.502 |
| ST-T abnormality | 17 (12.6) | 6 (14.3) | 11 (11.8) | 0.690 |
| Atrial arrhythmia | 6 (4.4) | 1 (2.4) | 5 (5.4) | 0.665 |
| LA enlargement | 1 (0.7) | - | 1 (1.1) | - |
| Ventricular arrhythmia | 7 (5.2) | 1 (2.4) | 6 (6.5) | 0.435 |
| Conduction abnormality | 3 (2.2) | 1 (2.4) | 2 (2.2) | - |
| Low limb lead voltage | 1 (0.7) | - | 1 (1.1) | - |
| Complex ECG abnormality | 37 (28.1) | 14 (33.3) | 23 (25.8) | 0.300 |
| **Echo abnormalities** | 27 (79.4), n=34 | 13 (92.9), n=14 | 14 (70.0), n=20 | 0.198 |
| Pericardial effusion | 2 (5.9) | 1 (7.1) | 1 (5.0) | - |
| LV diastolic dysfunction | 9 (26.5) | 2 (14.3) | 7 (35.0) | 0.108 |
| RV abnormality (PAH) | 1 (2.9) | - | 1 (5.0) | - |
| LA enlargement | 1 (2.9) | 1 (7.1) | - | 0.412 |
| Other complex abnormalities | 14 (41.2) | 9 (64.3) | 5 (25.0) | **0.022** |
| **CT/DR abnormalities (%)** | 221 (72.7) | 69 (71.9) | 152 (73.1) | 0.827 |

ECG: electrocardiogram; LA: left atrial, LV/RV: left/right ventricle; PAH: pulmonary arterial hypertension; CT/DR, Computed Tomography/ Digital Radiography.

**Table S2:** Multivariate Cox Regression Analysis on the Risk Factors Associated with Mortality in Patients With COVID-19 from symptom onset

| Factors | Univariate analysis | | Cox regression model | |
| --- | --- | --- | --- | --- |
|  | Hazard ratio (95% CI) | *P* value | Hazard ratio (95% CI) | *P* value |
| Age, > 65 years | 3.79 (2.32-6.20) | **< 0.001** | 2.22 (1.15-4.27) | **0.017** |
| Sex | 0.53 (0.34-0.82) | **0.005** |  |  |
| Hypertension | 2.05 (1.34-3.13) | **0.001** |  |  |
| COPD | 2.95 (1.64-5.32) | **< 0.001** | 2.43 (1.11-5.31) | **0.027** |
| Chronic heart disease | 2.16 (1.34-3.47) | **0.002** |  |  |
| Cerebrovascular disease | 3.10 (1.72-5.59) | **< 0.001** |  |  |
| Hs-TnI | 1.07 (1.05-1.10) | **< 0.001** | 3.33 (1.96-5.66) | **< 0.001** |
| CRP | 1.01 (1.01-1.02) | **< 0.001** | 1.01 (1.01-1.01) | **<0.001** |
| NT-proBNP on admission | 1.00 (1.00-1.00) | 0.082 |  |  |
| PCT | 1.00 (1.00-1.02) | 0.768 |  |  |
| D-dimer | 1.01 (1.00-1.01) | **0.003** |  |  |
| NCP types |  | **< 0.001** |  | **0.001** |
| severe-common | 3.89 (1.95-7.76) | **< 0.001** | 2.43 (1.05-5.67) | **0.039** |
| critically severe-common | 18.42 (9.06-37.41) | **< 0.001** | 5.40 (2.09-13.95) | **0.001** |

P-values by Cox regression analyses. PCT, CRP, and NT-proBNP on admission, D-dimer were performed as continuous variables. NT-proBNP, N-terminal pro-B-type natriuretic peptide; COPD: chronic obstructive pulmonary disease; PCT, Procalcitonin; NCP: novel coronary pneumonia; Hs-TnI: high-sensitivity troponin I.

**Figure Legends:**

**Fig. S1** CT presentation of patients with COVID-19.


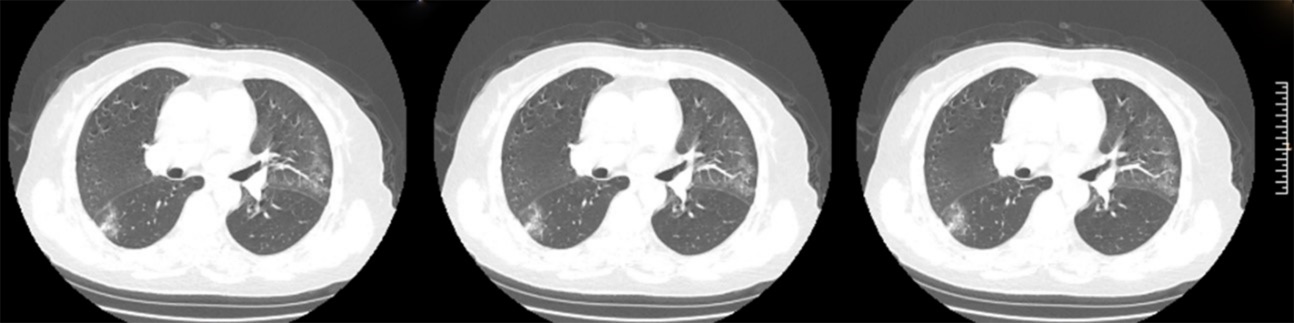


A older female patient was diagnosed with COVID-19, presenting with fever and shortness of breath at the onset. She had hypertension for 4 years. CT examination indicated multi-focal patch, and segmental ground glass opacities.
